# Role of Textural Analysis Parameters Derived from FDG PET/CT in Diagnosing Cardiac Sarcoidosis

**DOI:** 10.1055/s-0044-1788336

**Published:** 2024-07-12

**Authors:** Rutuja Kote, Mudalsha Ravina, Rangnath Thippanahalli Ganga, Satyajt Singh, Moulish Reddy, Pratheek Prasanth, Rohit Kote

**Affiliations:** 1 Department of Nuclear Medicine, All India Institute of Medical Sciences Raipur, Raipur, Chhattisgarh, India; 2 Department of Pulmonary Medicine, All India Institute of Medical Sciences Raipur, Raipur, Chhattisgarh, India; 3 Department of Cardiology, All India Institute of Medical Sciences Raipur, Raipur, Chhattisgarh, India; 4 Department of Computer Science, Indian Institute of Technology Jodhpur, Jodhpur, Rajasthan India

**Keywords:** artificial intelligence, cardiac, radiomics, sarcoidosis, textural analysis

## Abstract

**Introduction** Texture and radiomic analysis characterize the lesion's phenotype and evaluate its microenvironment in quantitative terms. The aim of this study was to investigate the role of textural features of 18F-fluorodeoxyglucose (18F-FDG) positron emission tomography–computed tomography (PET/CT) images in differentiating patients with cardiac sarcoidosis (CS) from patients with physiologic myocardial uptake.

**Methods** This is a retrospective, single-center study of 67 patients, 17 diagnosed CS patients, and 50 non-CS patients. These patients underwent FDG PET/CT for the diagnosis of CS. The non-CS group underwent 18F-FDG PET/CT for other oncological indications. The PET/CT images were then processed in a commercially available textural analysis software. Region of interest was drawn over primary tumor with a 40% threshold and was processed further to derive 92 textural and radiomic parameters. These parameters were then compared between the CS group and the non-CS group. Receiver operating characteristics (ROC) curves were used to identify cutoff values for textural features with a *p* -value < 0.05 for statistical significance. These parameters were then passed through a principle component analysis algorithm. Five different machine learning classifiers were then tested on the derived parameters.

**Results** A retrospective study of 67 patients, 17 diagnosed CS patients, and 50 non-CS patients, was done. Twelve textural analysis parameters were significant in differentiating between the CS group and the non-CS group. Cutoff values were calculated for these parameters according to the ROC curves. The parameters were Discretized_HISTO_Entropy, GLCM_Homogeneity, GLCM_Energy, GLRLM_LRE, GLRLM_LGRE, GLRLM_SRLGE, GLRLM_LRLGE, NGLDM_Coarseness, GLZLM_LZE, GLZLM_LGZE, GLZLM_SZLGE, and GLZLM_LZLGE. The gradient boosting classifier gave best results on these parameters with 85.71% accuracy and an F1 score of 0.86 (max 1.0) on both classes, indicating the classifier is performing well on both classes.

**Conclusion** Textural analysis parameters could successfully differentiate between the CS and non-CS groups noninvasively. Larger multicenter studies are needed for better clinical prognostication of these parameters.

## Introduction

18F-fluorodeoxyglucose (18F-FDG) positron emission tomography (PET) plays an important role in the diagnosis and assessment of cardiac sarcoidosis (CS). 1 Sarcoidosis is a systemic disorder, which affects multiple organs. Cardiac involvement is an important prognostic factor in sarcoidosis patients. Therefore, a definitive and accurate diagnosis is necessary. There are various methods for diagnosing and assessing the activity of CS, including objective and quantitative assessments using 18F-FDG PET/computed tomography (CT). 2

Various methods for evaluating FDG uptake in the diagnosis and management of CS have been reported. The most popular approaches include visual assessment, 2 semiquantitative analysis using standardized uptake value (SUV), 3 and volume-based analyses using cardiac metabolic volume (CMV) and cardiac metabolic activity (CMA). 4

In FDG PET/CT, a glucose analog is used as a tracer. It represents the lesion glycolytic activity. The most widely used parameter is SUVmax, the maximum SUV.

Many recent studies have concluded that the heterogeneity of the FDG uptake provides diagnostic and prognostic benefits in CS patients 5 6 7 8 9 10 .

The FDG uptake is variable at places due to necrosis, hypoxia, cell proliferation, and microvessel density. 11 More heterogeneous lesions are more aggressive and correlate with poorer outcomes.

Textural analysis characterizes tumor heterogeneity in the form of PET image-derived quantitative indices. It extracts meaningful quantitative parameters from two- or three-dimensional (3D) images. They allow for the quantification of tumor phenotypic characteristics. The textural analysis is based on the spatial arrangement and distribution of voxels in a volume of interest. 12

This study aims to evaluate the role of textural analysis in differentiating the CS group from the non-CS patients. Whether the myocardial FDG textural analysis could add diagnostic value beyond the standard FDG diagnostic indices in the diagnosis of CS was also analyzed.

## Materials and Methods

### Patients

In this retrospective single-center investigation, we prospectively analyzed patients diagnosed with CS. Seventeen patients without oral steroid treatment before the FDG PET/CT scan were included between October 2019 and December 2023.

For the control group (non-CS group), 50 consecutive patients who underwent an FDG PET/CT scan for evaluating malignant tumors in 2023 were retrospectively analyzed. All non-CS patients suffered from a malignant disease or had been treated for a malignant disease. The fasting duration was obtained from the patient interview record before FDG administration. Patients who were < 20 years old, patients whose imaging protocol was different, and patients without any uptake in the myocardium were excluded from the control group. In all patients, available clinical parameters, including age, sex, and histology, were recorded.

### Imaging Technique

All CS patients fasted overnight (for at least 18 hours) preceded by a low-carbohydrate diet, with less than 5 g of carbohydrate per meal. Note that 50 IU/kg heparin was administered to the CS patients 20 minutes before the FDG injection. Non-CS patients were instructed not to consume any food other than plain water for at least 6 hours before the time of injection of FDG, with no special dietary preparation such as a low-carbohydrate meal.

All the PET/CT scans were performed using a GE Discovery MI-DR PET/CT scanner for staging. The fasting blood glucose (FBG) was checked before the scan. If the glucose levels were in the normal fasting range, patients were injected with an 18F-FDG injection. The injections were done according to the weight of the patients. At 18F-FDG injection, the mean plasma glucose level was 100 mg/dL. CT from the brain to mid-thigh was performed before the PET scan using a 16-slice CT scanner. Whole-body PET was performed, covering an identical area to that covered by CT. Acquisition time was 1 to 2 minutes per bed position, with 7 to 8 bed positions. The obtained images were then exported to the textural analysis software.

### Image Analysis

The images were exported to commercially available textural analysis software. The FDG uptake pattern in the myocardium was visually interpreted in consensus by two board-certified nuclear medicine physicians. They were blinded to clinical, pathological, and other imaging information. The myocardium was delineated manually, and regions of interest (ROIs) were drawn over the myocardium. The ROI was then delineated with 40% thresholding. Then, the ROI was processed to obtain the textural indices. All the parameters were extracted from the delineated lesions.

### Standardized Uptake Value Analysis

Activity in a lesion is reported in terms of the SUVmax. SUVmax is the value of the most intense pixel in the ROI. This allows the exclusion of low counts from areas of necrosis adjacent to normal structures. SUVmean is an average of all counts in the ROI, which may be more representative because a spurious single hot area will not cause incorrect data to be recorded. SUVpeak is the average of the counts from a circular volume surrounding the hottest pixel. The SUVpeak may more accurately represent maximal tumor metabolism with a higher degree of statistical significance than the SUVmax. Metabolic tumor volume (MTV) refers to the metabolically active volume of the tumor. Total lesion glycolysis is the product of MTV and SUVmean. All these values are provided by the software automatically.

### Textural Analysis

Once the images are processed, the software provides different types of textual indices and matrices. There are three different types of textural features—first-order, second-order, and higher-order textural features. First-order textural features are statistics based on the gray-level distribution of the image but do not consider relative positions of gray levels. They quantify intensity variations between each voxel and its immediate neighbors. Second- and higher-order textural features consider relative positions of gray levels and therefore allow quantification of heterogeneity.

### First-Order Parameters

First-order parameters quantify intensity variations between each voxel and its immediate neighbors. These are intensity-based and histogram-based parameters. They include parameters like entropy, skewness, and energy.

*Entropy* reflects irregularity in the gray level. A completely random distribution would have very high entropy.

*Energy* reflects the uniformity of the distribution.

*Skewness* reflects the asymmetry of the gray-level distribution.

*Kurtosis* reflects the shape of the gray-level distribution relative to normal distribution.

### Second-Order Parameters

These are regional heterogeneity parameters. They are calculated through analysis at the level of groups of boxes and areas of various sizes and intensities. They include:

1. Gray level zone length matrix (GLZLM): It provides information on the size of homogeneous zones for each gray level in three dimensions. From this matrix, 11 textural indices can be computed. They depend on the size of the zone if it is a long zone or short zone and the level of intensity; if it is a low gray level or a high gray level.
2. Gray level run length matrix ( GLRLM): It gives the size of homogeneous runs for each gray level. The matrix is computed for the 13 different directions in 3D and for each of the 11 textural indices derived from the matrix. They depend on the size of the run if it is a long run or short run, and the level of intensity if it is a low gray level or a high gray level.

### Higher-Order Parameters

These parameters tell us about spatial interrelationships and frequency distributions of the gray levels. They include matrices like neighborhood gray level difference matrix and gray level cooccurrence matrix (GLCM).

1. Neighborhood gray level difference matrix (NGLDM): It corresponds to the difference of gray level between 1 voxel and its 26 neighbors in three dimensions. Three textural indices are computed from this matrix.
  i. NGLDM_Coarsness: Is the level of spatial rate of change in intensity.
  ii. NGLDM_Contrast: Is the intensity difference between neighboring regions.
  iii. NGLDM_Busyness: This is the spatial frequency of changes in intensity.
2. Gray level cooccurrence matrix (GLCM): It takes into account the arrangements of pairs of voxels to calculate textural indices. Six textural indices are computed from this matrix.
  i. GLCM_Homogeneity: Is the homogeneity of gray-level voxel pairs.
  ii. GLCM_Energy: Is the uniformity of gray-level voxel pairs.
  iii. GLCM_Contrast: Is the local variations in the GLCM.
  iv. GLCM_correlation: Is the linear dependency of gray levels in GLCM.
  v. GLCM_Entropy: Is the randomness of gray-level voxel pairs.
  vi. GLCM_Dissimiliarity: This is the variation of gray-level voxel pairs.

### Statistical Analysis

The analysis included 67 patients, 17 diagnosed CS patients and 50 non-CS patients. These patients underwent 18F-FDG PET/CT on a GE Discovery MI-DR PET/CT scanner. The images were processed in commercially available textural analysis software. ROI was drawn over the myocardium with a 40% threshold and was processed further to derive 92 textural and radiomic parameters. These parameters were then compared between the CS group and the non-CS group. Receiver operating characteristic (ROC) curves were used to identify the optimal cutoff values for the textural features with a *p* -value < 0.05 for statistical significance using statistical analysis software. Specificity and sensitivity (including 95% confidence intervals) for each of the studied parameters were derived using ROC curves measuring associated areas under the ROC curves (AUC). Textural results were compared with those of SUVmax and SUVmean for their ability to distinguish between the CS group and the non-CS group. These parameters were then passed through a principle component analysis algorithm. Five different machine learning classifiers were then tested on the derived parameters.

## Results

This study involved 67 patients, 17 diagnosed CS patients and 50 non-CS patients. For the non-CS group, patients without any cardiac uptake were excluded from the study. A total of 50 patients (62.5 ± 13.0 years old, 35 males) were included in the non-CS group. Non-CS patients included 9 patients suffering from malignant lymphoma, 2 from squamous cell carcinoma of unknown primary lesion, 9 from lung cancer, 6 from colon cancer, 5 from cervical cancer, 10 from breast cancer, and 9 from esophageal cancer. Eight patients had diabetes mellitus. Gender and age showed no significant differences between the CS and non-CS groups. However, the fasting period was longer and the FBG was lower for the CS group compared to the non-CS group.

All the patients' characteristics are described in Table 1 .

**Table 1:** Patient characteristics

Patient characteristics		Number
Sex	Male	40
	Female	27
Diabetes	Present	8
	Absent	59
Cardiac sarcoidosis		17
Non-cardiac sarcoidosis		50

By textural analysis, the AUC values were calculated for all the different parameters. Neither SUVmax nor SUVmean significantly differed between the CS group and the non-CS group.

**Fig. 1:**
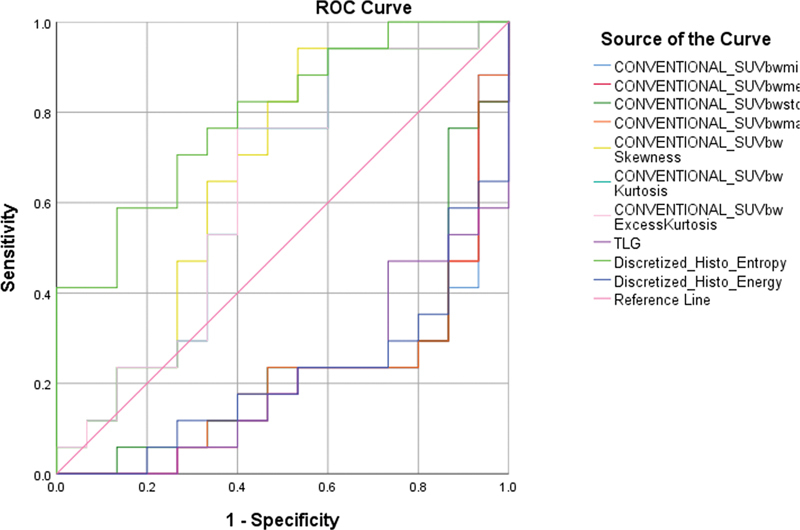
Receiver operating characteristics (ROC) analysis for first order parameters.

In the case of first-order parameters ( Fig. 1 ), discretized_Histo_Entropy was significant in differentiating between the CS and non-CS groups. The AUC value for this parameter was 0.79 and the cutoff was calculated to be 0.1092. The rest of the parameters were not significant to differentiate between the CS and non-CS groups. They had an AUC value below 0.7. Among the rest of the first-order parameters, SUV skewness, SUV kurtosis, and SUV excess kurtosis have a maximum AUC value of 0.63.

**Fig. 2:**
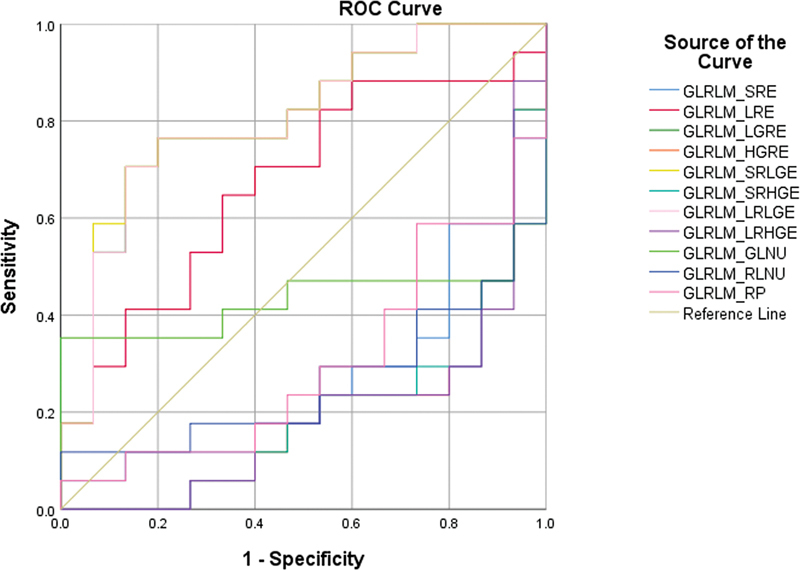
Receiver operating characteristics (ROC) analysis of gray level run length matrix (GLRLM) parameters.

**Fig. 3:**
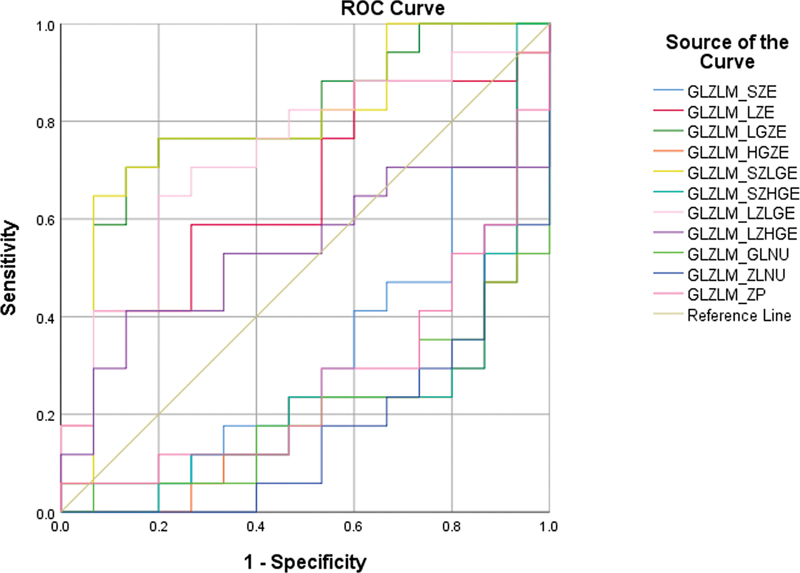
Receiver operating characteristics (ROC) analysis for gray level zone length matrix (GLZLM) parameters.

Among the second-order parameters, four parameters from the GLRLM matrix ( Fig. 2 ) and four parameters from the GLZLM matrix ( Fig. 3 ) were significant in differentiating the CS group from the non-CS group. They were GLRLM_LRE, GLRLM_LGRE, GLRLM_SRLGE, and GLRLM_LRLGE and GLZLM_LZE, GLZLM_LGZE, GLZLM_SZLGE, and GLZLM_LZLGE. The cutoffs were calculated for these parameters. The cutoffs are given in Table 2 , and the AUC values are described in Table 3 . These parameters tell about the distribution of the size of homogeneous zones for each gray level in three dimensions.

**Table 2:** Cutoff values for GLRLM and GLZLM matrix parameters

Sr. no.	Parameters	Cutoffs
1.	GLRLM_LRE	1.333
2.	GLRLM_LGRE	0.0041
3.	GLRLM_SRLGE	0.0037
4.	GLRLM_LRLGE	0.0060
5.	GLZLM_LZE	40.97
6.	GLZLM_LGZE	0.0039
7.	GLZLM_SZLGE	0.0020
8.	GLZLM_LZLGE	0.4192

Abbreviations: GLRLM, gray level run length matrix; GLZLM, gray level zone length matrix.

**Table 3:** AUC values for GLRLM and GLZLM matrix parameters

Sr. no.	Parameters	AUC values
1.	GLRLM_LRE	0.71
2.	GLRLM_LGRE	0.804
3.	GLRLM_SRLGE	0.808
4.	GLRLM_LRLGE	0.804
5.	GLZLM_LZE	0.794
6.	GLZLM_LGZE	0.792
7.	GLZLM_SZLGE	0.792
8.	GLZLM_LZLGE	0.729

Abbreviations: AUC, area under the curve; GLRLM, gray level run length matrix; GLZLM, gray level zone length matrix.

**Fig. 4:**
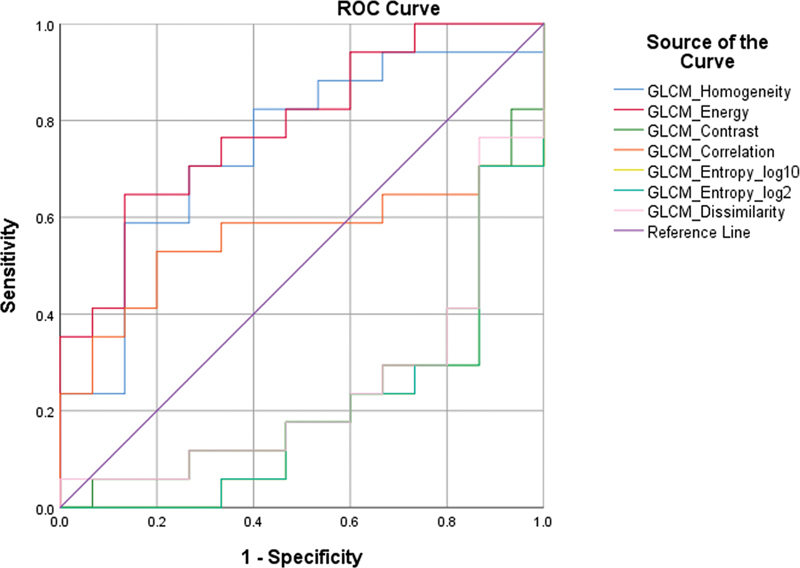
Receiver operating characteristics (ROC) analysis for gray level cooccurrence matrix (GLCM) parameters.

**Fig. 5:**
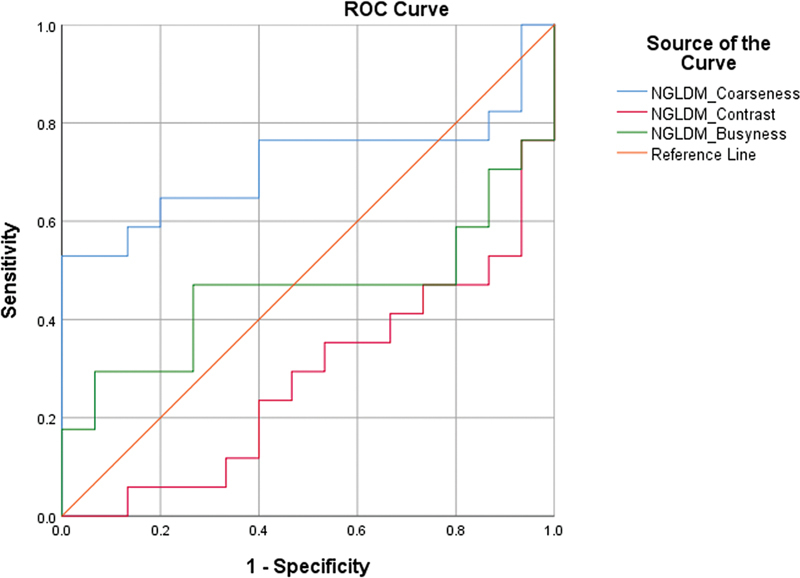
Receiver operating characteristics (ROC) analysis for neighborhood gray level difference matrix (NGLDM) parameters.

In the case of higher-order parameters ( Figs. 4 and 5 ), GLCM_homogeneity, GLCM_Energy, and NGLDM_Coarseness were significant in differentiating the CS and non-CS groups. GLCM_homogeneity represents the homogeneity of the tumor.

Other higher parameters like contrast, busyness, etc., in the NGLDM and GLCM matrix, were not statistically significant predictive factors of response.

The cutoffs are given in Table 4 , and the AUC values are described in Table 5 .

**Table 4:** Cutoff values for GLCM and NGLDM matrix parameters

Sr. no.	Parameters	Cutoff values
1.	GLCM_Homogeneity	0.399
2.	GLCM_Energy	0.0130
3.	NGLDM_Coarseness	0.0020

Abbreviations: GLCM, gray level cooccurrence matrix; NGLDM, neighborhood gray level difference matrix.

**Table 5:** AUC values for GLCM and NGLDM matrix parameters

Sr. no.	Parameters	AUC values
1.	GLCM_Homogeneity	0.745
2.	GLCM_Energy	0.788
3.	NGLDM_Coarseness	0.718

Abbreviations: AUC, area under the curve; GLCM, gray level cooccurrence matrix; NGLDM, neighborhood gray level difference matrix.

These parameters were then passed through a principle component analysis algorithm. Five different machine learning classifiers were then tested on the derived parameters. The gradient boosting classifier gave best results on these parameters with 85.71% accuracy and an F1 score of 0.86 (max 1.0) on both classes, indicating the classifier is performing well on both classes.

## Discussion

18F-FDG PET is crucial for the diagnosis, care, and treatment monitoring of CS patients. 13 In clinical practice, the SUVmax is a frequently used semiquantitative technique for making diagnoses, assessing disease activity, and tracking treatment response. 3 Nevertheless, because the SUVmax only represents one voxel, it is unable to fully capture the dispersion of a target lesion's metabolism. 14 The titration of immunosuppressive medication has been guided and cardiac events have been predicted by volume-based studies of FDG PET parameters including CMV and CMA, which have been employed as assessment instruments. 15 It can occasionally be challenging to discriminate between physiological and inflammatory lesions because physiological FDG uptake in the left ventricular wall is not limited to diffuse uptake. 16

Currently, increased interest is noted in the use of image-derived textural analysis parameters for the quantification of intratumor heterogeneity. The interpretation of images depends on the observer's education, expertise, and experience. The ability to identify diseases based only on qualitative criteria is strongly operator-dependent. 17 The need for more objective and quantitative evaluation of medial images has stimulated efforts to identify reliable imaging biomarkers with the development of a new research field called radiomics.

Radiomic and textural analysis allow for performing a whole-body assessment and characterizing lesions in a noninvasive way. Correlations between textural features and lesion phenotype have been described. 18 In the case of lung lesions, Miwa et al found that CT-derived morphological complexity and PET-derived intratumor heterogeneity evaluated by fractal analysis differ significantly between malignant and benign lesions. 19 According to Zhang et al, PET and PET/CT texture parameter models can improve the predictability of clear cell renal cell carcinoma Furhman nuclear grade. 20 Bianconi et al found that significant associations emerged between PET features, CT features, and histological type in non-small cell lung cancer. 21 Kuno et al found that CT texture analysis may be a noninvasive method of obtaining additional quantitative information to differentiate nodal metastases from disease-specific nodal reactivity in human immunodeficiency virus-positive patients with head and neck squamous cell carcinoma. 22

In CS patients, quantitative assessment of the variability of FDG uptake has demonstrated benefits for prognosis and diagnosis. 5

The myocardium's CS tissue exhibits heterogeneity on both macro- and microscopically. 23 When exploring intradisease heterogeneity based on the spatial distribution of uptake, FDG PET/CT is a useful noninvasive method. According to Sperry et al, the coefficient of variation was used to describe the heterogeneity of myocardial FDG uptake, and this gave a predictive predictor for unfavorable cardiac events. 24 Magnetic resonance imaging (MRI) has already been used to apply texture analysis to heart lesions. 25 Larroza et al attempted to use texture analysis on cine MRI and late gadolinium enhancement MRI of the heart. They concluded that texture analysis could be utilized to differentiate between myocardial infarction that is acute and that is chronic. 26

In this study, we assessed the potential role of textural indices in differentiating between the CS group and non-CS group patients.

## SUV-Related Parameters and First-Order Parameters

In this study, ROC analysis was performed on SUV-related parameters and first-order textural parameters. One parameter, Discretized_Histo_Entropy was significant in differentiating the CS group from the non-CS group. This parameter provides information on the asymmetry and uniformity of the image.

## Second-Order Parameters

In this work, the second-order and the higher-order parameters were significant in differentiating the CS group from the non-CS group.

The second-order parameters provide information on the size of homogeneous areas. Four parameters from GLRLM and four parameters from the GLZLM matrix were significant, with AUC values of more than 0.7.

## GLZLM Matrix

The GLZLM matrix is a second-order parameter. It quantifies gray-level zones in an image. A gray-level zone is defined as the number of connected voxels that share the same gray-level intensity. These are the regional heterogeneity parameters. In our study, four parameters from the GLZLM_matrix were significant in differentiating the CS group from the non-CS group. They were GLZLM_LZE, GLZLM_LGZE, GLZLM_SZLGE, and GLZLM_LZLGE. AUC value was more than 0.7 for these parameters.

GLZLM_LZE (Long Zone Emphasis): is a measure of the distribution of large area size zones, with a greater value indicative of larger size zones and more coarse textures.

GLZLM_LGZE (Low Gray Zone Emphasis): is a measure of the distribution of the low gray zones in the image.

GLZLM_SZLGE (Short Zone Low Gray Level Emphasis): measures the proportion in the image of the joint distribution of short-size zones with lower gray-level values.

GLZLM_LZLGE(Long Zone Low Gray Level Emphasis): measures the proportion in the image of the joint distribution of larger size zones with lower gray-level values.

These parameters quantify the coarseness of the image. They map the areas with low gray levels and assess their sizes and arrangements. The response of the lesion to the steroid therapy depends on these gray levels and their arrangements. The more variation in the texture and gray level of the tumor, the more aggressive the lesion would be.

## GLRLM Matrix

The GLRLM matrix is a second-order parameter. It quantifies gray level runs, which are defined as the length in a number of pixels of consecutive pixels that have the same gray level value. These are the regional heterogeneity parameters. In our study, four parameters from the GLRLM matrix were significant in differentiating the CS group from the non-CS group. They were GLRLM_LRE, GLRLM_LGRE, GLRLM_SRLGE, and GLRLM_LRLGE with an AUC value of more than 0.7.

GLRLM_LRE (Long Run Emphasis): is a measure of the distribution of long run lengths, with a greater value indicative of longer run lengths and more coarse structural textures.

Similar to the GLZLM matrix, the parameters from the GLRLM matrix quantify the heterogeneity of the tumor. The more heterogeneity, the more aggressive the lesion will be.

GLRLM_LGRE (Low Gray Run Emphasis): measures the distribution of low gray-level values, with a higher value indicating a greater concentration of low gray-level values in the image.

GLRLM_SRLGE (Short Run Low Gray Emphasis): measures the joint distribution of shorter run lengths with lower gray-level values.

GLRLM_LRLGE (Long Run Low Gray Emphasis): measures the joint distribution of long-run lengths with lower gray-level values.

## Higher-Order Parameters

Higher-order parameters provide information about spatial interrelationships and frequency distributions of the gray levels.

## GLCM Matrix

The GLCM takes into account the arrangements of pairs of voxels to calculate textural indices.

GLCM_Contrast is a measure of the local intensity variation. A larger value correlates with a greater disparity in intensity values among neighboring voxels.

GLCM_Correlation is a value between 0 (uncorrelated) and 1 (perfectly correlated), showing the linear dependency of gray-level values to their respective voxels in the GLCM.

GLCM_Homogeneity is the homogeneity of gray-level voxel pairs.

GLCM_Energy is a measure of homogeneous patterns in the image. A greater energy implies that there are more instances of intensity value pairs in the image that neighbor each other at higher frequencies.

Among the higher-order parameters, GLCM homogeneity and GLCM energy were significant in differentiating the CS group and the non-CS group.

## NGLDM Matrix

The NGLDM matrix quantifies the difference between a gray value and the average gray value of its neighbors.

NGLDM_Coarseness is a measure of the average difference between the center voxel and its neighborhood and is an indication of the spatial rate of change. A higher value indicates a lower spatial change rate and a locally more uniform texture.

NGLDM_Contrast measures the spatial intensity change but also depends on the overall gray-level dynamic range. Contrast is high when both the dynamic range and the spatial change rate are high, that is, an image with a large range of gray levels, with large changes between voxels and their neighborhood.

NGLDM_Busyness is a measure of the change from a pixel to its neighbor. A high value for busyness indicates a “busy” image, with rapid changes of intensity between pixels and their neighborhood.

In this study, NGLDM_Busyness was significant in differentiating the CS patients from the non-CS patients.

Therefore, this result indicated that the CS group and non-CS group had significantly different homogeneous uptake patterns.

Manabe et al concluded that LRE was a significant independent factor that could distinguish between CS and non-CS with high interoperator reproducibility and high diagnostic ability. 10 Our results confirmed the previous findings and provided some additional parameters to differentiate between the CS group and the non-CS group.

In summary, a single feature cannot be directly linked to a specific biological process. One could assume that a combination of textual parameters may be closely related to underlying physiological processes such as vascularization, perfusion, tumor aggressiveness, or hypoxia. 27 Therefore, textural features could be correlated to morphological phenotype.

## Limitations

The limitation of the present study is that it is retrospective, considering a relatively small patient cohort. Therefore, the potential of new image-derived indices characterizing lesion FDG distribution for differentiating CS from physiologic myocardial uptake needs to be validated by a prospective study on a larger patient cohort.

## Conclusion

In our study, the value of textual feature analysis was explored in the FDG PET scans for differentiating between CS and non-CS patients. Global metabolic features based on the intensity histogram were computed directly on the original image. Three orders of features were derived from the textual analysis—first order, second order, and higher order.

These features evaluated in this study highlighted lesion heterogeneity at a local and regional level characterized in several ways depending on the type of matrix used and the kind of feature computed on the matrix. In conclusion, textural parameters derived from 18F-FDG PET/CT can differentiate between the CS group and the non-CS group.
